# Estimated GFR reporting is not sufficient to allow detection of chronic kidney disease in an Italian regional hospital

**DOI:** 10.1186/1471-2369-10-24

**Published:** 2009-09-01

**Authors:** Giorgio Gentile, Maurizio Postorino, Raymond D Mooring, Luigi De Angelis, Valeria Maria Manfreda, Fabrizio Ruffini, Manuela Pioppo, Giuseppe Quintaliani

**Affiliations:** 1 Department of Internal Medicine, University of Perugia, Perugia, Italy; 2 Department of Nephrology and Dialysis, "Ospedali Riuniti" and CNR-IBIM, Reggio Calabria, Italy; 3 Analysis Made Easy, Ellenwood, GA, USA; 4 Shorter College, Atlanta, GA, USA; 5 Analysis Laboratory, Santa Maria della Misericordia Hospital, Perugia, Italy; 6 Department of Nephrology and Dialysis, Santa Maria della Misericordia Hospital, Perugia, Italy; 7 Finance and Administration, Santa Maria della Misericordia Hospital, Perugia, Italy

## Abstract

**Background:**

Chronic kidney disease (CKD) is an emerging worldwide problem. The lack of attention paid to kidney disease is well known and has been described in previous publications. However, little is known about the magnitude of the problem in highly specialized hospitals where serum creatinine values are used to estimate GFR values.

**Methods:**

We performed a cross-sectional evaluation of hospitalized adult patients who were admitted to the medical or surgical department of Santa Maria della Misericordia Hospital in 2007. Information regarding admissions was derived from a database. Our goal was to assess the prevalence of CKD (defined as an estimated glomerular filtration rate [eGFR] < 60 mL/min/1.73 m^2^) and detection of CKD using diagnostic codes (Classification of Diseases, Ninth Revision, Clinical Modification [ICD-9-CM]). To reduce the impact of acute renal failure on the study, the last eGFR obtained during hospitalization was the value used for analysis, and intensive care and nephrology unit admissions were excluded. We also excluded patients who had ICD-9-CM codes for renal replacement therapy, acute renal failure, and contrast administration listed as discharge diagnoses.

**Results:**

Of the 18,412 patients included in the study, 4,748 (25.8%) had reduced eGFRs, falling into the category of Kidney Disease Outcomes Quality Initiative (KDOQI) stage 3 (or higher) CKD. However, the diagnosis of CKD was only reported in 19% of these patients (904/4,748). It is therefore evident that there was a "gray area" corresponding to stage 3 CKD (eGFR 30-59 ml/min), in which most CKD diagnoses are missed. The ICD-9 code sensitivity for detecting CKD was significantly higher in patients with diabetes, hypertension, and cardiovascular disease (26.8%, 22.2%, and 23.7%, respectively) than in subjects without diabetes, hypertension, or cardiovascular disease (p < 0.001), but these values are low when the widely described relationship between such comorbidities and CKD is considered.

**Conclusion:**

Although CKD was common in this patient population at a large inpatient regional hospital, the low rates of CKD detection emphasize the primary role nephrologists must play in continued medical education, and the need for ongoing efforts to train physicians (particularly primary care providers) regarding eGFR interpretation and systematic screening for CKD in high-risk patients (i.e., the elderly, diabetics, hypertensives, and patients with CV disease).

## Background

Chronic kidney disease (CKD) represents a serious public health problem, as CKD rates are constantly rising all over the world [1-4]. Large population-based studies [2,5] have shown that 1 of every 10 adults is affected by CKD in the United States. This disease is defined by the National Kidney Foundation Kidney Disease Outcomes Quality Initiative (NKF KDOQI ™) as either kidney damage or decrease in glomerular filtration rate (GFR) persisting for at least three months, and is classified in five stages of increasing severity (KDOQI stages) [6]. CKD can be easily detected with simple biochemical tests, including the estimated glomerular filtration rate, which is calculated from serum creatinine values (eGFR) [7,8]. Identifying subjects with CKD (i.e., those with an eGFR <60 ml/min/1.73 m^2^, or KDOQI stage ≥ 3) is extremely important for two reasons. The first is that timely intervention can slow disease progression, thereby increasing the period of time that patients are free from dialysis, and reducing both risks for patients and costs to national health care systems[9,10]. The second is that patients with stage 4 or 5 CKD have a 2- to 4-fold greater risk of developing cardiovascular disease than age-matched subjects with normal kidney function [1,11-13], and it has been shown that early identification of CKD may reduce cardiovascular disease rates in these patients [14,15].

While the primary prevention of CKD is somewhat difficult, secondary prevention should theoretically be easier because it is related to numerous risk factors that are present in a large proportion of the population; these include diabetes, high blood pressure, cardiovascular disease, and other lifestyle factors that are relatively easy to identify. Secondary prevention, however, requires a timely diagnosis of CKD. Indeed, several previous studies [7,8] have suggested that an early diagnosis of CKD can be obtained from patients' eGFR, calculated using the Modification of Diet in Renal Disease (MDRD) formula [16-18].

The existence of tests for detecting a given disease does not mean that the disease will necessarily be identified. Ryan [19] demonstrated that as many as three-quarters of patients affected by CKD who were under the care of general practitioners in Great Britain had not been diagnosed. The situation in Italy is similar. One large Italian study demonstrated that only one of seven patients affected by CKD and under the care of general practitioners was correctly identified [20].

In order to simplify the identification of CKD and attract attention to this problem, many laboratories are now equipped with software that automatically calculates patients' eGFR using standardized formulas. Our hospital's laboratory (Santa Maria della Misericordia Hospital, Perugia, Italy) reports eGFR using the MDRD formula for all patients who have serum creatinine testing performed. The two values are listed one after the other in order to allow physicians to recognize immediately the association between the serum creatinine value and renal function. However, the extent to which this measure has succeeded at improving the rate of diagnosis of CKD remains unknown.

KDOQI guidelines define CKD as a decrease in GFR for a duration of at least three months. However, it is difficult to obtain a long-term follow-up for a large number of hospitalized patients. Therefore, despite the fact that a single evaluation of eGFR may overestimate the number of patients with CKD, we evaluated how often hospital physicians included CKD in patients' discharge diagnoses (by listing an ICD-9 code reflective of CKD), even for hospitalizations not directly related to kidney disease, when data regarding eGFR were available during the hospitalization.

## Methods

Data for this cross-sectional study were obtained by analyzing the records of the centralized analysis laboratory of Santa Maria della Misericordia Hospital in Perugia. The computerized system uses software developed by Bayer - SIEMENS that is based on Oracle and the LMX system. In order to maintain the integrity of laboratory data, the system sends the data to an external database that can be queried via the internet using software developed by TECNIDATA SrL.

Serum creatinine testing was carried out using ADVIAA 1800 (SIEMENS) equipment and was based on the reaction of picric acid with creatinine in an alkaline environment, as originally described by Jaffe [21]. Calibration of creatinine levels was performed by the SIEMENS method using High Performance Liquid Chromatography (HPLC). We queried the laboratory database to obtain all laboratory results of all patients admitted to Santa Maria della Misericordia Hospital between January 1^st ^and December 31^st ^2007. We excluded patients admitted to intensive care units or to the nephrology department. We obtained a file that contained a total of 107,569 serum creatinine values for 33,743 hospitalizations that occurred during 2007.

For patients who had multiple creatinine values for the same admission, we considered only the last creatinine value (i.e., the value obtained closest to the time of discharge, and therefore the value that would best approximate a patient's renal function at the time of discharge). We also excluded 1,452 admissions of pediatric patients (less than 15 years of age), 65 admissions for renal replacement therapy, and 6,795 admissions in which diagnoses were missing or the data were incomplete. The final file contained complete data on 25,431 hospitalizations (or discharges) of 18,412 patients. A total of 3,996 patients were admitted more than once during 2007, for a total of 11,015 admissions (3,996 first admissions and 7,019 consecutive admissions, mean number of admissions = 2.7 per patient).

For each patient included in the final analysis, we obtained the last measured serum creatinine value before admission and the associated eGFR, which was calculated using the appropriate MDRD formula [eGFR = 175 × (sCr.^-1.154 ^× age^-0.203^)]. This value is multiplied by 0.742 for females [7,8]. We also collected data regarding discharge diagnoses as documented by ICD-9 codes (note that at discharge, up to six separate diagnoses may be listed), discharge department, and age at discharge. Patients were classified according to Kidney Disease Outcomes Quality Initiative (KDOQI) stages and CKD was defined as the presence of CKD with KDOQI stage 3 or higher (a moderate or severe reduction in GFR or kidney failure).

We considered the ICD-9 codes listed in Table 1 to be reflective of the presence of kidney disease when listed as discharge diagnoses. When patients had additional ICD-9 codes for renal replacement therapy (i.e., v56A, v45B), acute renal failure (i.e., 584.5, 584.8, 584.9), or contrast procedures (i.e., 88.4×, 88.5×), they were excluded from the study. We obtained the codes we used from ICD9-CM-2002 (Italian Version), which was introduced into the Italian coding system on January 1, 2006.

**Table 1:** ICD-9-CM codes for kidney disorders*

ICD-9 Code	Diagnosis
**585**	Chronic kidney disease (CKD)

**586**	Renal failure, unspecified

*2002 version

The primary analysis sought to determine the sensitivity, specificity, positive likelihood ratio (LR), negative LR and post-test odds of ICD-9 diagnosis codes for the detection of chronic kidney disease. We did this in order to test the ability of administrative databases based on ICD-9 codes to accurately identify CKD when it is present, and in order to verify whether reduced eGFR levels drew attention to CKD independently from the cause of admission.

Sensitivity was defined as the proportion of patients with CKD who were correctly identified, and specificity as the proportion of patients without CKD who were correctly identified based on the ICD-9 codes listed at the time of discharge. The positive LR was calculated by dividing the sensitivity of the assay by the false positive rate (1-specificity). The negative LR was calculated by dividing the false negative rate (1-sensitivity) of the assay by its specificity [22]. LRs greater than 1 tell us a test result is more likely to occur among patients with the disease than among those without the disease; LRs less than 1 tell us a result is less likely to occur among patients with the disease than among patients without the disease. LRs of 10 or more usually "rule in" disease; LRs of 0.1 or less usually "rule out" disease. An LR of 1 is completely useless in ruling disease in or out [23]. The positive and negative LRs do not change as the underlying probability of disease changes, because the probability of disease is captured mostly in the prevalence term. The main term that contains prevalence information is the pre-test odds, which can be calculated as:

For this reason, pre-test odds are used to "weight" the positive and negative LR. The "weighted" positive and negative LR are mathematically equivalent to the post-test odds, that can be calculated as: Post-test odds = Pre-test odds × Likelihood Ratio. Therefore, the post-test odds incorporate information about the disease prevalence, the patient pool, and specific patient risk factors (pre-test odds), as well as information about the diagnostic test itself (the likelihood ratio). The post-test probability can be calculated as: Post-test probability = post-test odds/(post-test odds + 1). A LR greater than one produces a post-test probability which is higher than the original pre-test probability. A LR less than one produces a post-test probability which is lower than the original pre-test probability [24].

The inherent variation in test performance among patient subgroups, or spectrum effect [25], was assessed by comparing the sensitivity and specificity values of the assay for patients in two predetermined subgroups (e.g., age ≥ 70 years vs. < 70 years, males vs. females) using the *z *test. Additionally, we explored whether changing the eGFR threshold for CKD from the "true" value of < 60 ml/min to < 30 ml/min (stages 4 and 5 only) or even to < 15 ml/min (stage 5 only) was able to change ICD-9 performance. We did so in order to determine whether physicians are more likely to notice lower reported eGFR values when assigning ICD-9 codes at the time of discharge. Secondary analyses explored the correlation between multiple risk factors (such as diabetes, hypertension, and cardiovascular disease) and the prevalence of CKD. When an ICD-9 code for diabetes mellitus was present as one of the six discharge diagnoses, the patient was coded as diabetic ("1" code). Similarly, a patient was considered hypertensive (coded as "1") when an ICD-9 code for essential hypertension was present as one of the six discharge diagnoses. Cardiovascular disease was considered present (coded as "1") when any of the six discharge diagnoses contained an ICD-9 code corresponding to the following diseases: myocardial infarction (including subendocardial infarction), congestive heart failure, coronary heart disease (including angina), or stroke. Uni- and multivariate log-binomial regression analysis was conducted to determine the prevalence ratios for the outcome, CKD [26,27], using the following dichotomized variables: age (≥ 70 vs. < 70), sex (male vs. female), diabetes (yes/no), hypertension (yes/no), CV disease (yes/no), and department of admission (medical vs. surgical). For the purposes of the primary and secondary analyses, only the first admission was considered in order to avoid counting the same patients twice. Finally, for each patient with repeated admissions, the first and last admission were compared in order to assess test-retest reliability of reported eGFR values and inter-rater agreement of the ICD-9 diagnosis of CKD using the κ statistic and the phi coefficient [28]. For this study, we consider a kappa less than zero to indicate less than chance agreement, 0.01-0.20 slight agreement beyond chance, 0.21-0.40 fair agreement, 0.41-0.60 moderate agreement, 0.61-0.80 substantial agreement, and 0.81-0.99 almost perfect agreement [29]. The phi coefficient measures the correlation between two nominal variables and ranges from -1 to 1. This measure is similar to the correlation coefficient in its interpretation [30]. Descriptive statistics were assessed and are presented as frequencies, means, standard deviations, and confidence intervals. Groups were compared using the unpaired Student's t-test or the χ^2 ^test. For all analyses, a two-sided p-value of less than 0.05 was considered to indicate statistical significance. Statistical analysis was performed using SPSS 16.0 (SPSS Inc., Chicago, IL, USA) and STATA version 10.0 (StataCorp LP, College Station, Texas, USA).

## Results

The performance characteristics of ICD-9 codes for detecting CKD in various subgroups of patients admitted during 2007 are reported in [additional file 1]. Only data from the first admission were included for patients admitted multiple times in 2007.

Of the 18,412 patients included in the analysis, 52.7% were male. The mean age of included patients was 61.5 ± 19.8 (mean ± SD) years (range 15-99 years). Patients discharged from surgical departments were younger (61.7 ± 18.8 years) than patients discharged from medical departments (66.1 ± 17.6 years). Age (≥ 70 vs. < 70 years) did not affect test sensitivity, which was about 19% for both age groups (p = 0.86).

Medical comorbidities such as diabetes, hypertension, and CV disease associated with a risk of developing CKD, were associated with a statistically significant improvement in test sensitivity (26.8%, 22.2%, and 23.7% vs. 17.1%, 16.9% and 16.1% in subjects without diabetes, hypertension or CV disease, respectively; p < 0.001).

Admission to medical departments (internal medicine and its branches: oncology, endocrinology, gastroenterology, geriatrics, occupational medicine, hematology, infectious diseases, and cardiology) was associated with a statistically significant improvement in test sensitivity as compared to admission to surgical departments (general surgery and its branches: surgical oncology, thoracic surgery, digestive tract, urology, heart surgery, orthopedic surgery, vascular surgery, ophthalmology, ear, nose, and throat surgery, and neurosurgery) (17.4% vs. 13.0%, p = 0.001).

In male patients, the sensitivity of ICD-9 codes for detecting the presence of CKD was 28.4%, as compared to 12% in female patients (p < 0.001).

The overall sensitivity of ICD-9 codes for detecting patients with CKD (stage 3 or higher) was low at only 19%. However, when the eGFR threshold for CKD was lowered from the "true" value of less than 60 ml/min to 30 ml/min (stage 4 or 5 only) or even 15 ml/min (stage 5 only), a striking increase in ICD-9 sensitivity was found (55.7% and 70.6%, respectively).

The overall prevalence of CKD was high (25.8%, 95% CI 25.1-26.4), and it was even higher in "high-risk" patients such as diabetics (41.3%, 95% CI 39.3-43.3), hypertensives (36.9%, 95% CI 35.6-38.2) and patients with CV disease (43.9%, 95% CI 42.4-45.4), as compared to lower-risk patients (p < 0.001). The prevalence of CKD was higher in females than in males (31.1% vs. 21%, p < 0.001), which is consistent with previous reports [31].

Table 2 shows the results of uni- and multivariate analyses of each of the clinical variables that were included in the study; age ≥ 70 years, female sex, and admission to medical departments as well as the presence of diabetes, hypertension, and CV disease were all associated with a statistically significant increase in the estimated prevalence ratios for CKD, both in the unadjusted and in the adjusted models (p < 0.001). This relationship was particularly strong in the elderly (adjusted PR 3.41, 95% CI 3.2-3.63, p < 0.001).

**Table 2:** Prevalence ratios of CKD*

Covariates PR	Estimated PR (unadjusted)	95% CI	P	Estimated (adjusted)	95% CI	P
**Age ≥ 70 vs. age <70**	4.11	3.88-4.37	<0.001	3.41	3.20-3.63	<0.001

**Female vs. male**	1.48	1.41-1.55	<0.001	1.45	1.39-1.51	<0.001

**Diabetes vs. no diabetes**	1.75	1.66-1.85	<0.001	1.21	1.16-1.27	<0.001

**Hypertension vs**. **no hypertension**	1.72	1.64-1.80	<0.001	1.23	1.18-1.28	<0.001

**CV disease vs**. **no CV disease**	2.14	2.04-2.24	<0.001	1.41	1.35-1.47	<0.001

**Admission to medical** **vs. surgical department**	1.52	1.44-1.60	<0.001	1.09	1.03-1.14	0.001

*Values obtained from uni- and multivariate log-binomial regression models

PR: prevalence ratio; CI: confidence interval

When analyzing patients with multiple admissions, the agreement was moderate between the first and last admission in terms of CKD status as assessed by reported eGFR (κ: 0.429, p < 0.001; phi: 0.522, p < 0.001). The inter-rater agreement for ICD-9 diagnosis of CKD was also moderate (κ: 0.493, p < 0.001; phi:0.497, p < 0.001). Of course, inter-rater agreement does not necessarily imply a correct evaluation: physicians could be using incorrect evaluation schemes.

Finally, when all 25,431 discharges are considered, an ICD-9 diagnosis of kidney disorder was the main diagnosis in 267 cases, the second diagnosis in 356 cases, the third diagnosis in 342 cases, the fourth diagnosis in 330 cases, the fifth diagnosis in 257 cases, and the sixth diagnosis in 133 cases, for a total of 1,685 cases (6.6% of discharges).

Table 3 lists the KDOQI stage of the 25,431 hospitalizations considered in the study. A total of 9,132 discharges (35.9% of the total, corresponding to 4,748 patients with CKD) had stage 3 or higher CKD, but a correct ICD-9 diagnosis of CKD was reported in only 1,579/9,132 (16%) cases. On the other hand, ICD-9 diagnosis of CKD was incorrectly reported in only 106/16,299 (0.65%) admissions, with the renal function of these patients falling into stages 1 or 2 CKD as determined by eGFR reporting.

**Table 3:** Number of admissions with or without the ICD-9 diagnosis of CKD at discharge, stratified by KDOQI stage

KDOQI Stage				
**ICD-9 diagnosis**	**3**	**4**	**5**	**All admissions with KDOQI stage ≥ 3**

**No CKD**	6,838 (90.5%)	550 (7.3%)	165 (2,2%)	7,553
**CKD**	793 (50.2%)	490 (31.1%)	296 (18.7%)	1,579

KDOQI: Kidney Disease Outcomes Quality Initiative

## Discussion

The prevalence of CKD is increasing worldwide due to the aging of the general population and the increasing prevalence of diabetes, hypertension, and CV disease [3]. Among the 18,412 patients hospitalized during 2007, the overall prevalence of CKD was high (about 25%). In particular, nearly half of patients older than 70 years of age had CKD. The prevalence of CKD in patients with CV disease, diabetes and hypertension was only slightly lower than the prevalence in older individuals (44%, 41%, and 37%, respectively). CKD has been shown to be an independent risk factor for adverse cardiovascular events and mortality. In fact, for each CKD patient who lives long enough to develop end-stage renal disease requiring dialysis, several patients have already had fatal or non-fatal CV events [1,32]. Therefore, identifying patients with CKD has become a major health issue. However, identification of kidney disease does not often happen early in the course of the disease, not only because it progresses asymptomatically until kidney function is severely compromised, but also because the reported "normal" range for serum creatinine values does not take into consideration patients' age and anthropomorphic characteristics. To solve this problem, a number of standardized formulas for estimating patients' creatinine clearance [33] or glomerular filtration rate [7,8] have been developed.

The health information systems that are currently in place in various health organizations [34] use the MDRD formula to calculate an eGFR value for each patient who has serum creatinine levels tested in order to overcome problems associated with the variability of "normal" creatinine values and their interpretation by physicians.

The lack of attention paid to kidney disease by physicians is well known from the literature. This study is the first one to quantify this phenomenon in a highly specialized hospital where patient eGFRs are calculated in a standardized fashion each time the serum creatinine is evaluated. Thus, all physicians who request serum creatinine values are always informed about that patient's renal function, and it is up to the physician to place the proper emphasis on it.

At least theoretically, being aware of this value and correctly interpreting it should help with the diagnosis of CKD [35], as timely identification is extremely important for slowing progression and reducing the associated increase in cardiovascular risk [36,37]. In sharp contrast with this hypothesis, the present study highlights that in 80% of hospitalizations in which there was documented evidence of renal failure by eGFR, CKD was not listed as a final ICD-9 code diagnosis, thus underscoring the pitfalls associated with this approach [38-40]. Notably, we found that while only 19% of patients with stage 3 or higher CKD (eGFR < 60 ml/min) received a correct ICD-9 diagnosis of kidney failure, this percentage rose to 56% in patients with stage 4 or 5 CKD (eGFR < 30 ml/min). Although diagnosing half of patients with advanced kidney disease based on available laboratory data should not be considered a "good" result, it is evident that ICD-9 performance improved considerably in this subgroup of patients. There may be several explanations for this. Perhaps physicians *do *actually take into account lower values of reported eGFR (i.e., values <30 ml/min) when assigning ICD-9 codes at discharge, but fail to recognize moderate CKD (stage 3) as a clinical problem. Alternatively, it is possible that they use creatinine values when assigning an ICD-9 diagnosis of CKD, because creatinine values are usually only elevated above the "normal" range when eGFR falls below 30 ml/min; for higher values of eGFR, creatinine values remain "normal", and therefore an ICD-9 diagnosis of CKD may remain overlooked. Whatever the cause, our study highlights the presence of a "gray area", corresponding to stage 3 CKD, in which most of the diagnoses are missed. In particular, when considering the first admissions of 18,412 patients admitted during 2007, 4,096 had an eGFR between 30 and 59 ml/min, but only 537 (13.1%) correctly received a diagnosis of chronic kidney disease. Thus, only one in eight patients with stage 3 CKD received an ICD-9 diagnosis of kidney failure. This is of great concern when considering the importance of early diagnosis not only in reducing the burden of kidney disease, but also in preventing the adverse effects of inadequate drug dosing or inappropriate exposure to nephrotoxic agents.

Another important point is the very low detection rate of CKD in the elderly that we observed in this study. Although the estimated prevalence ratio for CKD was 3.41 (3.20-3.63, p < 0.001) in patients ≥ 70 years of age as compared to patients <70 years of age, the ICD-9 sensitivity for detection of CKD was similar between the two groups (19% vs. 19.2%, p = 0.68), indicating that physicians failed to pay the necessary attention to renal function when evaluating higher-risk elderly patients.

On the other hand, detection of CKD through ICD-9 codes was significantly better in patients with diabetes mellitus, hypertension, or CV disease as compared to patients without diabetes, hypertension, or CV disease (sensitivity values: 26.8%, 22.2%, and 23.7%, vs. 17.1%, 16.9, and 16.1%, respectively; p < 0.001), although these values are still low when considering the widely described relationship between such comorbidities and CKD.

A statistically significant difference in the detection of CKD was also evident in patients admitted in medical departments as compared to surgical departments (17.4% vs. 13.0%, p = 0.001). It is possible that medical specialists are slightly more careful about evaluating eGFR at the time of discharge than surgical specialists. However, this phenomenon likely only has a small amount of clinical relevance given the magnitude of the problem.

The performance of ICD-9 codes in detecting CKD varied significantly between females and males (sensitivity: 12% vs. 28.4%, p < 0.001). We are unable to find any obvious explanation for this performance variation at the present time and are planning further analyses of our data to further explore the spectrum effect of ICD-9 performance.

Our study has several limitations. First, the diagnosis of chronic kidney disease was based on a single determination of serum creatinine level and eGFR [20,41] because our database was created using administrative data about a large number of hospitalized patients, and because nearly every hospitalization lasted less than three months. Consequently, we may have overestimated the number of patients with chronic kidney disease. However, the risk of overestimating CKD is counterbalanced by the use of a single centralized laboratory and by the availability of serum creatinine calibration[42,43], allowing the reduction of misclassification bias. Secondly, although eGFR has been widely used to diagnose CKD in hospitalized patients [44] with a wide spectrum of pathologies, including acute coronary syndromes [45], congestive heart failure [46] and stroke [47], equations for estimated GFR perform better for healthy, stable patients than for acutely ill, hospitalized patients [48]. To reduce the impact of acute renal failure on the study, the last eGFR for hospitalization was analyzed, intensive care and nephrology unit admissions were excluded, and patients whose discharge forms included ICD-9-CM code diagnoses, renal replacement therapy, acute renal failure, or contrast administration were excluded from the analysis.

## Conclusion

Because administrative information such as hospital discharge data is often used for surveillance purposes [49], underestimation of CKD among hospitalized patients may lead to a further reduced awareness of kidney disease among public health professionals, family medicine program directors and, ultimately, among general practitioners. It is important to note that nephrologists are consultants, and that they should not and cannot see all patients in order to diagnose CKD. Thus, the responsibility for the primary diagnosis of CKD lies in the hands of general practitioners and other specialists. However, an analysis based on the National Health and Nutrition Examination Surveys (NANHES III) and Medicare databases showed that CKD care is suboptimal [50], and two recent online surveys [51,52] suggest that primary care providers [53] and internal medicine residents may be not familiar with KDOQI guidelines. The presence of chronic renal failure is often considered an additional piece of information that has little effect on the general clinical assessment of patients, as if the presence of CKD does not modify patient prognosis and does not warrant intervention.

It therefore seems that the best approach to the problem of the under-diagnosis of CKD is to ensure that all health care professionals, both generalists and specialists, understand the importance of the early diagnosis of kidney disease. Physicians should be made especially aware that older patients and patients with diabetes, hypertension, or CV disease should be systematically screened for the presence of chronic kidney disease. This message could be easily transmitted through public health programs [54]. It is hoped that ICD-9 performance in detecting CKD will improve over time, in part due to the introduction of newer and more accurate coding systems such as ICD-9-CM-2007.

In conclusion, our study underscores the importance of the primary role that nephrologists have in the education of healthcare professionals, particularly primary care providers [55,56]. This will help to call attention to CKD, a pathology whose impact on public health is enormous and is rapidly rising.

## Competing interests

The authors declare that they have no competing interests.

## Authors' contributions

GG, GQ, MP and VMM made substantial contributions to the conception and design of the study, and were involved in drafting the manuscript. RDM, FR, MP and FDA substantially contributed to the acquisition, analysis and interpretation of data. All authors read and approved the final manuscript.

## Pre-publication history

The pre-publication history for this paper can be accessed here:

http://www.biomedcentral.com/1471-2369/10/24/prepub

## Supplementary Material

Additional file 1 **Performance characteristics of ICD-9 codes for detecting chronic kidney disease (stage 3 or higher) among various subgroups of patients admitted during 2007 (n = 18,412)**. The data provided represent the performance characteristics of ICD-9 codes for detecting CKD in various subgroups of patients. Click here for file
