# Supplementary material for: Estimated GFR reporting is not sufficient to allow detection of chronic kidney disease in an Italian regional hospital
Source: BMC Nephrol. 2009 Sep 1;10:24. doi: 10.1186/1471-2369-10-24 (PMC2749028; doi:10.1186/1471-2369-10-24)
Supplement: Additional file 1 — Performance characteristics of ICD-9 codes for detecting chronic kidney disease (stage 3 or higher) among various subgroups of patients admitted during 2007 (n = 18,412). The data provided represent the performance characteristics of ICD-9 codes for detecting CKD in various subgroups of patients. [file 1471-2369-10-24-S1.doc]

| ***Factor*** | ***Prevalence of CKD % (95% CI)*** | ***P valueº*** | ***Sensitivity % (95% CI)*** | ***P value †*** | ***Specificity % (95% CI)*** | ***P value‡*** | ***PLR (95% CI)*** | ***NLR (95%CI)*** | ***PO + (95% CI)*** | ***P value§*** | | ***PO- (95% CI)*** | ***P value§*** |  |
| --- | --- | --- | --- | --- | --- | --- | --- | --- | --- | --- | --- | --- | --- | --- |
| **Age ≥70 (n=8,009)** | 45.1 (44.0-46.2) | <0.001 | 19.0 (17.7-20.3) | 0.68 | 99.0 (98.7-99.3) | <0.001 | 18.16 (13.51-24.39) | 0.82 (0.81-0.83) | 14.89 (11.01-20.14) | | <0.001 | 0.67 (0.50-0.91) | <0.001 | |
| **Age <70 (n=10,403)** | 10.9 (10.3-11.5) |  | 19.2 (16.9-21.5) |  | 99.6 (99.5-99.8) |  | 52.39 (36.70-74.79) | 0.81 (0.79-0.83) | 6.44 (4.46-9.30) | |  | 0.10 (0.07-0.14) |  |  |
| **Females (n=8,706)** | 31.1 (30.1-32.1) | <0.001 | 12.0 (10.8-13.2) | <0.001 | 99.7 (99.6-99.8) | <0.001 | 37.91 (23.92-60.07) | 0.88 (0.87-0.89) | 17.10 (10.74-27.23) | | <0.001 | 0.40 (0.25-0.63) | <0.001 | |
| **Males (n=9,706)** | 21.0 (20.2-21.8) |  | 28.4 (26.4-30.4) |  | 99.2 (99.0-99.4) |  | 35.65 (27.50-46.20) | 0.72 (0.70-0.74) | 9.49 (7.25-12.43) | |  | 0.19 (0.15-0.25) |  |  |
| **Diabetes (n=2,323)** | 41.3 (39.3-43.3) | <0.001 | 26.8 (24.0-29.6) | <0.001 | 99.0 (98.5-99.6) | 0.028 | 28.12 (16.21-48.79) | 0.74 (0.71-0.77) | 19.77 (11.24-34.77) | | <0.001 | 0.52 (0.30-0.91) | <0.001 | |
| **No diabetes (n=16,089)** | 23.6 (22.9-24.2) |  | 17.1 (15.9-18.3) |  | 99.5 (99.3-99.6) |  | 31.35 (24.44-40.21) | 0.83 (0.82-0.85) | 9.66 (7.49-12.46) | |  | 0.26 (0.20-0.33) |  |  |
| **Hypertension (n=5,157)** | 36.9 (35.6-38.2) | <0.001 | 22.2 (20.3-24.0) | <0.001 | 98.9 (98.5-99.2) | <0.001 | 19.50 (14.00-27.16) | 0.79 (0.77-0.81) | 11.40 (8.10-16.05) | | <0.001 | 0.46 (0.33-0.65) | <0.001 | |
| **No Hypertension (n=13,255)** | 21.5 (20.8-22.2) |  | 16.9 (15.6-18.3) |  | 99.6 (99.5-99.7) |  | 41.02 (30.11-55.87) | 0.83 (0.82-0.85) | 11.21 (8.18-15.36) | |  | 0.23 (0.17-0.31) |  |  |
| **Admission to surgical department (n=6,767)*** | 20.1 (19.1-21.0) | <0.001 | 13.0 (11.2-14.7) | <0.001 | 99.5 (99.3-99.7) | 0.95 | 24.15 (16.38-35.61) | 0.88 (0.86-0.89) | 6.07 (4.08-9.03) | | <0.001 | 0.22 (0.15-0.33) | <0.001 | |
| **Admission to medical department (n=9,891)*** | 30.6 (29.7-31.6) |  | 17.4 (16.1-18.9) |  | 99.5 (99.3-99.7) |  | 34.14 (24.32-47.94) | 0.83 (0.82-0.84) | 15.09 (10.68-21.30) | |  | 0.37 (0.26-0.52) |  |  |
| **CV disease (n=4,133)** | 43.9 (42.4-45.4) | <0.001 | 23.7 (21.8-25.6) | <0.001 | 99.0 (98.6-99.4) | <0.001 | 22.93 (15.27-34.44) | 0.77 (0.75-0.79) | 17.96 (11.84-27.23) | | <0.001 | 0.60 (0.40-0.91) | <0.001 | |
| **No CV disease (n=14,279)** | 20.5 (19.9-21.2) |  | 16.1 (14.8-17.5) |  | 99.5 (99.4-99.6) |  | 32.67 (24.84-42.97) | 0.84 (0.83-0.86) | 8.45 (6.48-11.18) | |  | 0.22 (0.16-0.29) |  |  |
| **Overall ¥ (n=18,412)** | 25.8 (25.1-26.4) |  | 19.0 (17.9-20.2) |  | 99.4 (99.3-99.5) |  | 32.52 (25.94-40.77) | 0.81 (0.80-0.83) | 11.31 (9.03-14.19) | |  | 0.28 (0.27-0.29) |  | |
| **CKD+: eGFR <30 ml/min** | NA |  | 55.7 (51.8-59.4) |  | 96.6 (96.3-96.9) |  | NA | NA | NA | |  | NA |  | |
| **CKD+: eGFR <15 ml/min** | NA |  | 70.6 (63.9-76.6) |  | 95.4 (95.1-95.7) |  | NA | NA | NA | |  | NA |  | |

ICD-9: International Classification of Diseases Version 9; CI: confidence interval; PLR: positive likelihood ratio; NLR: negative likelihood ratio; PO +: post-test odds for a positive result; PO -: post-test odds for a negative result CV: cardiovascular

**º** P value for comparison of prevalences in the two subgroups

**†** P value for comparison of sensitivities in the two subgroups

**‡** P value for comparison of specificities in the two subgroups

**§** P value for the 2-sample proportion test

***** excludes 1,754 patients admitted to other departments

¥ Threshold for CKD +: eGFR <60 ml/min
